# Supplementary material for: Identification and functional analysis of non-coding regulatory small RNA FenSr3 in Bacillus amyloliquefaciens LPB-18
Source: PeerJ. 2023 May 15;11:e15236. doi: 10.7717/peerj.15236 (PMC10194069; doi:10.7717/peerj.15236)
Supplement: Supplemental Information 4 [file peerj-11-15236-s004.zip › KO/CK-vs-T1_map/map01522.html]

KEGG PATHWAY: Endocrine resistance - Reference pathway


|  |  |
| --- | --- |
| **Endocrine resistance - Reference pathway** |  |

[
Pathway menu
| Organism menu
| Pathway entry
| Show description
| User data mapping
]

Endocrine therapy is a key treatment strategy to control or eradicate hormone-responsive breast cancer. The most commonly used endocrine therapy agents are selective estrogen receptor modulators (SERMs, e.g. tamoxifen), estrogen synthesis inhibitors (e.g. aromatase inhibitors (AIs) such as anastrozole, letrozole, and exemestane), and selective estrogen receptor down-regulators (SERDs, e.g. fulvestrant). However, resistance to these agents has become a major clinical obstacle. Mechanisms of endocrine resistance include loss of ER-alpha expression, altered expression of coactivators or coregulators that play a critical role in ER-mediated gene transcription, ligand-independent growth factor signaling cascades that activate kinases and ER-phosphorylation, altered availability of active tamoxifen metabolites regulated by drug-metabolizing enzymes, such as CYP2D6, and deregulation of the cell cycle and apoptotic machinery.

|  |  |  |
| --- | --- | --- |
| Reference pathway | 184% 150% 122% 100% 82% 67% 55% | 图片下载 |
